# Dual Regulation of *Bacillus subtilis kinB* Gene Encoding a Sporulation Trigger by SinR through Transcription Repression and Positive Stringent Transcription Control

**DOI:** 10.3389/fmicb.2017.02502

**Published:** 2017-12-13

**Authors:** Yasutaro Fujita, Mitsuo Ogura, Satomi Nii, Kazutake Hirooka

**Affiliations:** ^1^Institute of Oceanic Research and Development, Tokai University, Shizuoka, Japan; ^2^Department of Biotechnology, Faculty of Life Science and Biotechnology, Fukuyama University, Fukuyama, Japan

**Keywords:** sporulation, biofilm formation, stringent transcription control, transcription initiation, SinR transcription regulator, RNA polymerase, decoyinine

## Abstract

It is known that transcription of *kinB* encoding a trigger for *Bacillus subtilis* sporulation is under repression by SinR, a master repressor of biofilm formation, and under positive stringent transcription control depending on the adenine species at the transcription initiation nucleotide (nt). Deletion and base substitution analyses of the *kinB* promoter (P*_kinB_*) region using *lacZ* fusions indicated that either a 5-nt deletion (Δ5, nt -61/-57, +1 is the transcription initiation nt) or the substitution of G at nt -45 with A (G-45A) relieved *kinB* repression. Thus, we found a pair of SinR-binding consensus sequences (GTTCTYT; Y is T or C) in an inverted orientation (SinR-1) between nt -57/-42, which is most likely a SinR-binding site for *kinB* repression. This relief from SinR repression likely requires SinI, an antagonist of SinR. Surprisingly, we found that SinR is essential for positive stringent transcription control of P*_kinB_*. Electrophoretic mobility shift assay (EMSA) analysis indicated that SinR bound not only to SinR-1 but also to SinR-2 (nt -29/-8) consisting of another pair of SinR consensus sequences in a tandem repeat arrangement; the two sequences partially overlap the ‘-35’ and ‘-10’ regions of P*_kinB_*. Introduction of base substitutions (T-27C C-26T) in the upstream consensus sequence of SinR-2 affected positive stringent transcription control of P*_kinB_*, suggesting that SinR binding to SinR-2 likely causes this positive control. EMSA also implied that RNA polymerase and SinR are possibly bound together to SinR-2 to form a transcription initiation complex for *kinB* transcription. Thus, it was suggested in this work that derepression of *kinB* from SinR repression by SinI induced by Spo0A∼P and occurrence of SinR-dependent positive stringent transcription control of *kinB* might induce effective sporulation cooperatively, implying an intimate interplay by stringent response, sporulation, and biofilm formation.

## Introduction

In *Bacillus subtilis*, entry into the sporulation pathway is governed by a member of the response regulator family of transcription factors known as Spo0A (Hoch, 1993). Spo0A is indirectly phosphorylated by a multicomponent phosphorelay system involving at least two kinases called KinA and KinB (Stephenson and Hoch, 2002). An increased level of phosphorylated Spo0A (Spo0A∼P) results in repression of *abrB* transcription (Strauch et al., 1990), leading to derepression of transcription of the *σH* (*spo0H*) gene encoding σ^H^. *kinA* is transcribed by RNA polymerase (RNAP) possessing σ^H^ (Predich et al., 1992), but *kinB* is transcribed by RNAP possessing σ^A^ (Trach and Hoch, 1993; Dartois et al., 1996). Hence, *kinB* transcribed by σ^A^-RNAP is supposed to be a trigger gene for sporulation rather than *kinA*.

Expression of the *kinA* and *kinB* genes is under positive stringent transcription control (Tojo et al., 2013). Their expression is induced upon amino acid starvation through GDP 3′-diphosphate (ppGpp) inhibition of GMP kinase (Kriel et al., 2012) or by the addition of decoyinine, a GMP synthase inhibitor (Mitani et al., 1977; Tojo et al., 2013), resulting in the reciprocal change of a GTP decrease and an ATP increase (Ochi et al., 1981; Tojo et al., 2010). The transcription initiation nucleotide (nt) of stringent promoters P*_kinA_*, P*_kinB_* and P*_ilvB_* (P*_ilv-leu_*) under positive stringent transcription control is the adenine species; *ilvB* is the first gene of the *ilv-leu* operon for branched-chain amino acid synthesis (Krásný et al., 2008; Tojo et al., 2008, 2013). In contrast, the transcription initiation nt of stringent genes such as *ptsG* and *pdhA* for glucose catabolism under negative stringent transcription control is the guanine species (Tojo et al., 2010). It is likely that occurrence of both the positive and negative stringent transcription controls causes the *B. subtilis* cell to enter the sporulation phase (Fujita et al., 2012; Tojo et al., 2013).

The *sinR* gene was originally isolated as a sporulation inhibition (sin) gene in multiple copies (Gaur et al., 1986). SinR represses transcription from the Spo0A∼P-dependent promoters of sporulation genes such as *spoIIA* and *spoIIG* (Cervin et al., 1998). Moreover, transcription of *kinB* was found to be repressed by SinR on *lacZ*-fusion analysis (Dartois et al., 1996). Furthermore, the SinR repressor is the master regulator of the formation of a biofilm, a natural lifestyle for most bacteria formed on natural and artificial surfaces (Kearns et al., 2005; Stewart and Franklin, 2008). The wild-type *B. subtilis* secretes exopolysaccharides (EPSs) and proteins to form an extracellular matrix for building the biofilm (Stewart and Franklin, 2008; Vlamakis et al., 2013). The extracellular matrices are composed of EPSs synthesized from the gene products of the 15-gene *epsA*-*O* operon, TasA protein fibers, and the BslA surface layer protein (Vlamakis et al., 2013). SinR is one of the major regulators of the genes required for biofilm formation. SinR binds to the promoter regions of the *epsA-O* and *tapA-sipW-tasA* operons to repress their expression (Kearns et al., 2005; Chu et al., 2006). The consensus DNA binding sequence for SinR comprises a 7-bp pyrimidine-rich sequence (GTTCTYT, with Y representing an unspecified pyrimidine base), which can be found in an inverted and tandem repeat orientation/arrangement and in a monomer state at SinR operator sites (Kearns et al., 2005; Chu et al., 2006; Colledge et al., 2011). The direct interaction of amino acid residues of SinR with bases of its consensus sequences in an inverted repeat orientation was visualized in the crystal structure of the complex of SinR with operator DNA of the *eps* promoter (Newman et al., 2013). SlrR is a protein homologous to SinR. SlrR binding to SinR inhibits the DNA-binding activity of SinR, and *slrR* expression itself is repressed by SinR (Kobayashi, 2008; Chai et al., 2010). Thus, these proteins form a double-negative feedback loop. The SinR antagonist SinI determines which protein is dominant in this loop through protein–protein interaction with SinR (Bai et al., 1993; Chai et al., 2008; Chu et al., 2008). *sinI* expression is transcriptionally induced by Spo0A∼P (Shafikhani et al., 2002), which is a master regulator of sporulation (Chai et al., 2008; Lopez et al., 2009). It was recently reported that post-transcriptionally regulated heterogeneous expression of SinR is important for the differentiation of cells present in a biofilm (Ogura, 2016).

In this work, we identified a pair of SinR consensus sequences in an inverted orientation (SinR-1) between nt -57/-42 (+1 is transcription initiation nt) as a SinR-binding site for *kinB* repression. Unexpectedly, we found that SinR is essential for positive stringent transcription control of P*_kinB_*. Electrophoretic mobility shift assay (EMSA) analysis indicated that SinR bound not only to SinR-1 but also to SinR-2 consisting of another pair of SinR consensus sequences in a tandem repeat arrangement (nt -29/-8) that partially overlap the ‘-35’ and ‘-10’ regions, respectively, which is likely involved in positive stringent transcription control of P*_kinB_*.

## Materials and Methods

### Bacterial Strains and Their Construction

The *B. subtilis* strains used in this work are listed in **Table 1**. To construct transcriptional promoter-*lacZ* fusion strains of *kinB*, P*_kinB_* regions comprising nt -75/+10, -75/+10 [with base substitution of A at nt +1 with G (A+1G)], -65/+10, -85/+10, -95/+10, -75/+10 [with 5-nt deletion (Δ5) (-61/-57)], -75/+10 [with 10-nt deletion (Δ10) (-64/-55)], and -75/+10 (with base substitution of G at -45 with A (G-45A) and Δ5] were amplified using the primer pairs of F75c/R10c1, F75c/R93, F90/R10c1, F92/R10c1, F82/R10c1, F95/R10c2, F96/R10c2, and F17/R17 (Supplementary Table S2-1), respectively, and DNA of strain 168 as a template. The PCR products were trimmed with XbaI and BamHI, and then ligated with the XbaI-BamHI arm of plasmid pCRE-test2 (Miwa and Fujita, 2001). The ligated DNAs were used for transformation of *Escherichia coli* strain DH5α to ampicillin-resistance (50 μg/ml) on Luria-Bertani (LB) medium plates (Sambrook and Russell, 2001). The correct construction of the fusions in the resulting plasmids was confirmed by DNA sequencing. The plasmids carrying the P*_kinB_* regions with and without a base substitution and (or) deletion were linearized with PstI, and then used for double-crossover transformation of strain 168 to chloramphenicol-resistance (5 μg/ml) on tryptose blood agar base (Difco) with 10 mM glucose (TBABG) plates, which produced strains FU1191 P*_kinB_* (-75/+10), FU1193 P*_kinB_* (-75/+10 A+1G), FU1190 P*_kinB_* (-65/+10), FU1192 P*_kinB_* (-85/+10), FU1182 P*_kinB_* (-95/+10), FU1195 P*_kinB_* (-75/+10 Δ5), FU1196 P*_kinB_* (-75/+10 Δ10), and FU1217 P*_kinB_* (-75/+10 G-45A Δ5), respectively.

**Table 1:** *Bacillus subtilis* strains used in this work.

Strain	Genotype	Reference
168	*trpC2*	Anagnostopoulos and Spizizen, 1961
FU1115	*trpC2 amyE*::[*cat* P*_kinB_* (-55/+10)-*lacZ*]	Tojo et al., 2013
FU1116	*trpC2 amyE*::[*cat* P*_kinB_* (-55/+10 A+1G)-*lacZ*]	Tojo et al., 2013
FU1191	*trpC2 amyE*::[*cat* P*_kinB_* (-75/+10)-*lacZ*]	This work
FU1193	*trpC2 amyE*::[*cat* P*_kinB_* (-75/+10 A+1G)-*lacZ*]	This work
FU1190	*trpC2 amyE*::[*cat* P*_kinB_* (-65/+10)-*lacZ*]	This work
FU1192	*trpC2 amyE*::[*cat* P*_kinB_* (-85/+10)-*lacZ*]	This work
FU1182	*trpC2 amyE*::[*cat* P*_kinB_* (-95/+10)-*lacZ*]	This work
FU1195	*trpC2 amyE*::[*cat* P*_kinB_* (-75/+10 Δ5)-*lacZ*]	This work
FU1196	*trpC2 amyE*::[*cat* P*_kinB_* (-75/+10 Δ10)-*lacZ*]	This work
FU1204	*trpC2 ΔsinR::erm*	This work
FU1206	*trpC2 ΔsinR::erm amyE::[cat* P_kinB_ (-55/+10)-*lacZ*]	This work
FU1210	*trpC2 ΔsinR::erm amyE::[cat* P_kinB_ (-75/+10)-*lacZ*]	This work
FU1216	*trpC2 amyE*::[*cat* P*_kinB_* (-75/+10 G-45A)-*lacZ*]	This work
FU1217	*trpC2 amyE*::[*cat* P*_kinB_* (-75/+10 G-45A Δ5)-*lacZ*]	This work
FU1218	*trpC2 ΔsinR::erm amyE::[cat* P_kinB_ (-75/+10 G-45*A)-lacZ]*	This work
FU1219	*trpC2 ΔsinR::erm amyE::[cat* P_kinB_ (-75/+10 G-45A *Δ*5) *-lacZ]*	This work
FU1224	*trpC2 ΔsinR::erm amyE::[cat* P_kinB_ (*-75/+*10*)Δ5-lacZ]*	This work
FU1225	*trpC2 ΔsinI::spc*	This work
FU1226	*trpC2 ΔslrR::tc*	This work
FU1230	*trpC2 ΔsinI::spc amyE::[cat P_kinB_* (-75/+10)-*lacZ*]	This work
FU1231	*trpC2 ΔslrR::tc amyE::[cat P_kinB_* (-75/+10)-*lacZ*]	This work
FU1237	*trpC2 ΔsinI::spc amyE::[cat* P*_kinB_* (-55/+10)-*lacZ*]	This work
FU1238	*trpC2 ΔslrR::tc amyE::[cat* P*_kinB_* (-55/+10)-*lacZ*]	This work
FU1241	*trpC2 amyE*::[*cat* P*_kinB_* (-55/+10 A-17G)*-lacZ*]	This work
FU1242	*trpC2 amyE*::[*cat* P*_kinB_* (-55/+10 G-16A)*-lacZ*]	This work
FU1243	*trpC2 amyE*::[*cat* P*_kinB_* (-55/+10 T-15C)*-lacZ*]	This work
FU1244	*trpC2 amyE*::[*cat* P*_kinB_* (-55/+10 G-14A)*-lacZ*]	This work
FU1245	*trpC2 amyE*::[*cat* P*_kinB_* (-55/+10 T-20C T-19C)*-lacZ*]	This work
FU1246	*trpC2 amyE*::[*cat* P*_kinB_* (-55/+10 T-18C A-17G)*-lacZ*]	This work
FU1247	*trpC2 amyE*::[*cat* P*_kinB_* (-55/+10 G-16A T-15C)*-lacZ*]	This work
FU1248	*trpC2 amyE*::[*cat* P*_kinB_* (-55/+10 C-26T T-25C)*-lacZ*]	This work
FU1249	*trpC2 amyE*::[*cat* P*_kinB_* (-55/+10 T-27C C-26T)*-lacZ*]	This work
ASK2102	*trpC2 rpoC*::pMUTinHis (Em^r^) *sigB Δ2* (Cm^r^) *sigH Δ*HB (Km^r^*)sigW Δ*HB (Sp^r^)	Yano et al., 2011


To construct strains FU1216 P*_kinB_* (-75/+10 G-45A), FU1241 P*_kinB_* (-55/+10 A-17G), FU1242 P*_kinB_* (-55/+10 G-16A), FU1243 P*_kinB_* (-55/+10 T-15C), FU1244 P*_kinB_* (-55/+10 G-14A), FU1245 P*_kinB_* (-55/+10 T-20C T-19C), FU1246 P*_kinB_* (-55/+10 T-18C A-17G), FU1247 P*_kinB_* (-55/+10 G-16A T-15C), FU1248 P*_kinB_* (-55/+10 C-26T T-25C), and FU1249 P*_kinB_* (-55/+10 T-27C C-26T), the upstream and downstream parts of the P*_kinB_* region (nt -75/+10) and the P*_kinB_* region (nt -55/+10) were separately amplified with the respective two primer pairs F16a/R16b and F16c/R10c1, F55c/R41b and F41c/R10c3, F55c/R42b and F42c/R10c3, F55c/R43b and F43c/R10c3, F55c/R44b and F44c/R10c3, F55c/R45b and F45c/R10c3, F55c/R46b and F46c/R10c3, F55c/R47b and F47c/R10c3, F55c/R48b and F48c/R10c3, and F55c/R49b and F49c/R10c3 for FU1216, FU1241, FU1242, FU1243, FU1244, FU1245, FU1246, FU1247, FU1248, and FU1249 (Supplementary Table S2-1) using chromosomal DNA of strains 168 as a template for FU1216 and chromosomal DNA of strain FU1115 P*_kinB_* (-55/+10) as a template for FU1241 to FU1249. Next, the respective two PCR products were mixed, and extension reactions were carried out without any primer. PCR with the resultant fragment as a template and a primer pair (F16a/R10c1 for FU1216, or F55c/R10c3 for FU1241 to FU1249)(Supplementary Table S2-1) was performed to amplify the combined DNA fragment, which was then trimmed with XbaI and BamHI, and cloned into plasmid pCRE-test2 (Miwa and Fujita, 2001) in *E. coli* strain DH5α, and the constructed plasmids were used for transformation of strain 168, as described above, resulting in strains FU1216, and FU1241 to FU1249.

Strain FU1204 (*ΔsinR*::*erm*) was constructed as follows. The regions upstream and downstream of the *sinR* gene were firstly amplified by PCR using DNA of strain 168 as a template, and primer pairs F04a/F04b and F04e/F04f, respectively. The *erm* cassette was amplified by PCR using DNA of plasmid pMUTIN2 (Yoshida et al., 2000) as a template, and primer pair F04c/F04d. Secondly, recombinant PCR involving primer pair F04a/F04f and three PCR fragments resulted in a PCR product covering the region upstream of *sinR*, the *erm* gene, and the region downstream of *sinR*. The resultant recombinant PCR product was used to transform strain 168 to erythromycin-resistance (0.3 μg/ml) on TBABG plates to produce strain FU1204. Strains FU1206, FU1210, FU1218, FU1219, and FU1224, which carry *ΔsinR*::*erm* and each of the *lacZ* fusions, were obtained by transformation of FU1115, FU1191, FU1216, FU1217, and FU1195 with DNA of strain FU1204 to erythromycin-resistance, respectively.

Strains FU1225 (*ΔsinI*::*spc*) and FU1226 (*ΔslrR*::*tc*) were obtained by transformation of strain 168 with DNAs of strain NCIB3610 carrying *ΔsinI*::*spc* (Ogura, 2016) and strain 168 carrying *ΔslrR*::*tc* (Ogura et al., 2014) to resistance to spectinomycin (60 μg/ml) and tetracycline (10 μg/ml) on TBABG plates, respectively. Strains FU1230, FU1237, FU1231, and FU1238, which carry *ΔsinI*::*spc* or *ΔslrR*::*tc*, and each of the *lacZ* fusions, were obtained by transformation of strains FU1191, and FU1115 [P*_kinB_* (-55/+10)] with DNAs of strain FU1225 or FU1226.

### Cell Cultivation and β-Galactosidase (β-Gal) Assaying

The *lacZ*-fusion strains were grown at 30°C overnight on TBABG plates containing the appropriate antibiotic(s); chloramphenicol (5 μg/ml), erythromycin (0.3 μg/ml), spectinomycin (60 μg/ml), and (or) tetracycline (10 μg/ml). The cells were inoculated with an optical density at 600 nm (OD_600_) of 0.1 in 50 ml of a nutrient sporulation medium (NSMP) (Fujita and Freese, 1981), and then cultivated. Then, 1 ml aliquots of the culture were withdrawn at 1-h intervals, and the β-Gal activity in crude cell extracts was measured spectrophotometrically, as described previously (Yoshida et al., 2000). The cells were also inoculated into 50 ml of a minimal sporulation medium containing 25 mM glucose and 50 μg/ml tryptophan (S6) (Fujita and Freese, 1981). (In the case of the inoculation of the *ΔsinR*, *ΔsinI,* and *ΔslrR* strains into S6 medium, the cells were first cultivated in LB medium before inoculation.) When the cells reached an OD_600_ of 0.5, 15 ml each culture was distributed into two flasks, and decoyinine was added to one flask to give a final concentration of 500 μg/ml (18 mM). Before and after decoyinine addition, 1-ml aliquots of the culture were withdrawn at 30-min intervals, and the β-Gal activity was measured.

### Sporulation Percentage Measurement

The titers of viable cells (V) and spores (S) that were heat-resistant (75°C for 20 min), for the cultures of strains 168 and FU1204 (*ΔsinR*), were measured to obtain the sporulation percentages (S/V x 100) at T0 and T20 (0 and 20 h after entry into the stationary cell phase during sporulation in NSMP). The sporulation percentages for S6 cultures at 0 and 10 h after decoyinine addition (T0 and T10) were also measured.

### Purification of SinR and RNAP

SinR was purified from *E. coli* RL4220, a BL21(DE3) derivative producing SinR (Kearns et al., 2005; Ogura et al., 2014), according to the method described previously, except for the use of a French pressure cell to prepare cell extracts (Chai et al., 2010). RNAP was purified from *B. subtilis* ASK2102 cells as described previously (Yano et al., 2011). The His tag was removed from His-SinR with biotinylated thrombin protease. SinR was dialyzed against dialysis buffer [10 mM Tris-Cl, 200 mM NaCl, 1 mM EDTA, 0.3 mM dithiothreitol (DTT), and 50% glycerol, pH 8.0]. His-RNAP was dialyzed against 10 mM Tris-Cl, 150 mM NaCl, and 30% glycerol, pH 8.0. The proteins were stored at -20°C.

### EMSA Analysis

The PCR primers and template DNA used for preparing biotinylated probes are shown in Supplementary Tables S1, S2-2. Site-directed mutagenesis of the probes was performed using an oligonucleotide-based PCR method as described previously (Ogura and Tanaka, 1996). For EMSA, appropriate amounts of SinR and (or) RNAP were incubated for 15 min at 28°C with a probe (20 fmol) in 16 μl of a reaction mixture (15 mM Tris-Cl, 4 mM MOPS-KOH, 15 mM KCl, 50 mM NaCl, 0.8 mM MgCl_2_, 0.6 mM DTT, and 12.5% glycerol, pH 7.8) containing 1 μg of poly(dI-dC) (GE Healthcare). After the addition of 2 μl of loading buffer [40% glycerol, 1× TBE (89 mM Tris-borate, and 2 mM EDTA, pH 8), 2 μg/ml bromophenol blue], the samples were applied onto a polyacrylamide gel, and electrophoresis was performed in 0.1× TBE buffer at 4°C. The method used for the detection of biotin-labeled DNA was described previously (Ogura and Tanaka, 1996).

Most EMSAs were performed with the gradient of the SinR concentration. Not a few critical EMSAs were duplicated.

## Results

### *kinB* Transcription and Its Regulation

**FIGURE 1:**
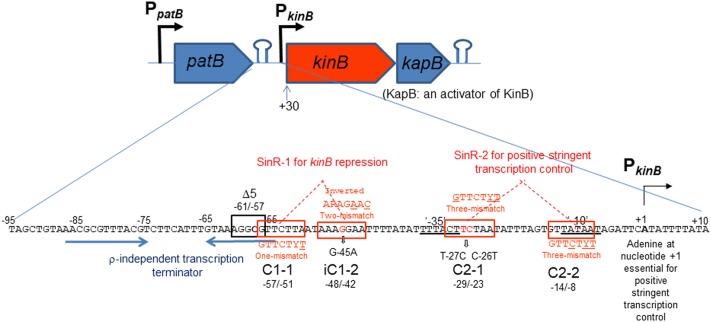
Schematic representation of the *patB-kinB-kapB* region of *B. subtilis*. Promoters (P*_patB_* and P*_kinB_*) and two stem-loop structures are shown. The *kinB* gene is cotranscribed with the *kapB* gene encoding an activator of KinB. This *kinB-kapB* transcription terminates at the stem-loop structure (ρ-independent terminator) downstream of *kapB*. *patB* transcription from P*_patB_* terminates at the stem-loop structure downstream of *patB*, which is indicated by two blue horizontal arrows. SinR-binding site 1 (SinR-1) consisting a pair of SinR consensus sequences (GTTCTYT) in an inverted orientation (C1-1, nt –57/–51 and iC1-2, nt –48/–42, boxed in red), which carries one mismatch and two mismatches to the consensus sequence (the mismatched nt are underlined), respectively. SinR-1 is involved in *kinB* repression. The Δ5 deletion (boxed in black, nt –61/–57) and G-45A substitution suppressed the repression. The ‘–35’ and ‘–10’ regions of P*_kinB_* are doubly underlined. SinR-binding site 2 (SinR-2) consisting of a pair of SinR consensus sequences in a tandem orientation (C2-1, nt –29/–23 and C2-2, nt –14/–8), both carrying three-mismatches to the consensus sequence. The T-27C C-26T substitution partially affected positive stringent transcription control of P*_kinB_*. SinR-2 is likely involved in positive stringent transcription control. The adenine species at transcription initiation nt (+1) is required for positive stringent transcription control to occur (Krásný et al., 2008; Tojo et al., 2010, 2013).

The *kinB* gene encoding one of the two major sensor kinases (KinA and KinB) of the phosphorelay system that phosphorylates Spo0A was identified, and its transcription was examined (Trach and Hoch, 1993). The *kinB* gene is transcribed from the σ^A^-dependent promoter, which starts from adenine (nt +1) (Trach and Hoch, 1993) (**Figure 1**). It is co-transcribed with *kapB* encoding a lipoprotein involved in autophosphorylation of KinB and phosphorylation of Spo0F (Dartois et al., 1997). An ρ-independent transcription terminator was found downstream of *kapB*, which presumably results in the *kinB-kapB* transcript. The *patB-*encoding aminotransferase is located immediately upstream of *kinB*. Another ρ-independent transcription terminator was found downstream of the *patB* gene, suggesting that the read-through of *patB* transcription is blocked. It was communicated in SubtiWiki 2.0^1^ (Michna et al., 2016) that the efficient blockage at the transcription terminator actually occurred. *kinB* transcription was reported to be repressed by SinR (Dartois et al., 1996). It was reported to be presumably repressed by AbrB (Strauch, 1995) and CodY (Molle et al., 2003). Recently, *kinB* expression was found to be under positive stringent transcription control (Tojo et al., 2013), that is, it is positively regulated upon stringent conditions such as amino acid starvation or on the addition of decoyinine, an inhibitor of GMP synthase, which induces stringent transcription control as well as sporulation. The positive stringent transcription control is strictly dependent on the adenine species at the transcription initiation nt, as described for *kinB* transcription (Tojo et al., 2013). However, *kinB* expression was not regulated by CodY or AbrB, at least as observed when examined by use of an *lacZ* fusion with the P*_kinB_* region (nt -55/+10)(Tojo et al., 2013)_._ To determine if the CodY- or AbrB-binding site is located outside of this region, we attempted to fuse a larger P*_kinB_* region with *lacZ* to yield the largest P*_kinB_* -*lacZ* fusion carrying P*_kinB_* (nt -95/+120); the larger fragment including the *patB* gene upstream of *kinB* could not be cloned to plasmid pCRE-test2, presumably because *patB* is harmful in its multiple copy state in *E. coli*. No significant difference in *lacZ* expression by the largest *lacZ*-fusion strain was observed in the wild-type, *ΔcodY*, and *ΔabrB* genetic backgrounds, on cultivation in NSMP or S6 medium with and without decoyinine (data not shown), suggesting that the CodY- and AbrB-binding sites that affect P*_kinB_* are unlikely to be located in the P*_kinB_* region (nt -95/+120). This finding implied that *kinB* expression might not be directly regulated by AbrB and CodY.

**FIGURE 2:**
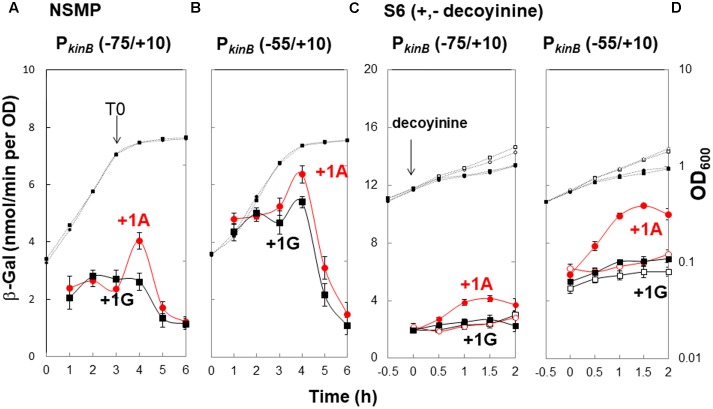
Requirement of the adenine species at the transcription initiation base for positive stringent transcription control of P*_kinB_*. The P*_kinB_* regions of nt –75/+10 and –55/+10 were fused with *lacZ* to yield strains FU1191 P*_kinB_* (–75/+10) and FU1115 P*_kinB_* (–55/+10). The adenine at nt +1 was replaced with a guanine to yield strains FU1193 P*_kinB_* (–75/+10 A+1G) and FU1116 P*_kinB_* (–55/+10 A+1G). The synthesis of β-Gal encoded by *lacZ* in strains FU1191 and FU1115, and strains FU1193 and FU1116 was monitored during sporulation in a nutrient sporulation medium, NSMP **(A,B)**, and after addition of decoyinine to the culture in minimal medium, S6 **(C,D)**. Circles and squares indicate the P*_kinB_* with adenine and guanine at nt +1, respectively. β-Gal synthesis during sporulation in NSMP was indicated by closed symbols. In the case of S6 medium, closed and open symbols indicate with and without addition of decoyinine, respectively. Large and small symbols denote β-Gal activity and OD_600_, respectively. In all Figures of β-Gal monitoring, the standard deviations of the average β-Gal activity values from the multiple replicates are indicated by error bars (one experiment gives two activity values at an indicated time); tiny error bars are invisible due to their overlap with the symbols. In the case of β-Gal monitoring shown in **(A,B)**, the experiments were performed with triple replicates.

To confirm that positive stringent transcription control of P*_kinB_* during sporulation in nutrient NSMP medium and upon decoyinine addition in minimal S6 medium is dependent on the adenine species at the transcription initiation nt, we constructed *lacZ* fusion strains with the P*_kinB_* region (nt -75/+10) carrying adenine and guanine at the transcription initiation nt, and β-Gal synthesis was monitored during sporulation of the constructed strains, P*_kinB_* (-75/+10) and P*_kinB_* (-75/+10 A+1G), together with the previously constructed strains, P*_kinB_* (-55/+10) and P*_kinB_* (-55/+10 A+1G) (Tojo et al., 2013) (**Figure 2**). The positive stringent transcription control was clearly observed in strains P*_kinB_* (-75/+10) and P*_kinB_* (-55/+10) for both sporulation in NSMP medium and decoyinine-induced sporulation in S6 medium, that is, some enhancement around T0.5 for sporulation in NSMP, and roughly a 1.5-fold increase after decoyinine addition, respectively (**Figure 2**). But, this positive control was not observed for strains P*_kinB_* (-75/+10 A+1G) and P*_kinB_* (-55/+10 A+1G). These results clearly confirmed that positive stringent transcription of P*_kinB_* depends on the adenine species at the transcription initiation nt (+1). Furthermore, the basal level of β-Gal synthesis was somewhat repressed in strains P*_kinB_* (-75/+10) and P*_kinB_* (-75/+10 A+1G) in comparison with that in strains P*_kinB_* (-55/+10) and P*_kinB_* (-55/+10 A+1G) for both sporulation in NSMP medium and decoyinine–induced sporulation in S6 medium (**Figure 2**), implying that the P*_kinB_* region (nt -75/-55) might possess a binding site or part of one for a transcription repressor.

In addition, it was notable that the positive stringent transcription control only partially contributed to enhancement of *kinB* transcription for sporulation in NSMP in contrast to a large contribution to it for decoyinine-induced sporulation in S6.

### Truncation and Deletion Analysis of the P*_kinB_* Region to Identify a Repressor-Binding Site

**FIGURE 3:**
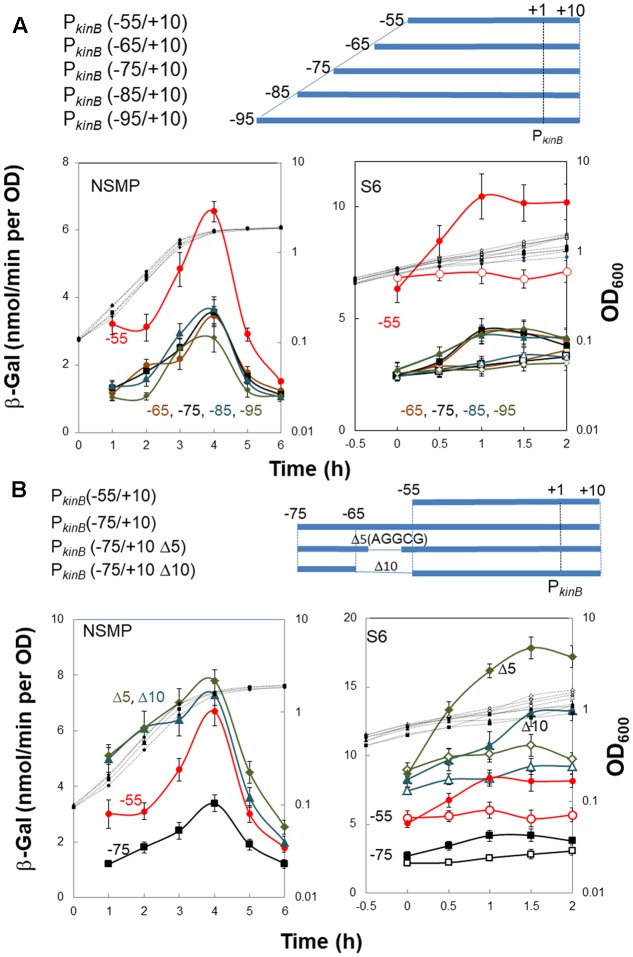
Truncation and deletion analyses of the P*_kinB_* region to identify a repressor-binding site. **(A)** Truncation analysis. β-Gal synthesis in strains FU1115 P*_kinB_* (–55/+10) (red circles), FU1190 P*_kinB_* (–65/+10) (brown circles), FU 1191 P*_kinB_* (–75/+10) (squares), FU1192 P*_kinB_* (–85/+10) (triangles), and FU1182 P*_kinB_* (–95/+10) (diamonds) was monitored during sporulation in NSMP medium and after addition of decoyinine to S6 medium. **(B)** Inner deletion analysis. β-Gal synthesis in strains FU1115 P*_kinB_* (–55/+10) (circles), FU 1191 P*_kinB_* (–75/+10) (squares), FU1195 P*_kinB_* (–75/+10 Δ5)(triangles), and FU1196 P*_kinB_* (–75/+10 Δ10) (diamonds) was monitored during sporulation in NSMP medium and after addition of decoyinine to S6 medium.

To localize a repressor-binding site in the P*_kinB_* region (nt -95/-55), we constructed a successive series of *lacZ*-fused P*_kinB_* truncation derivatives [P*_kinB_* (-95/+10), P*_kinB_* (-85/+10), P*_kinB_* (-75/+10), P*_kinB_* (-65/+10), and P*_kinB_* (-55/+10)]. When β-Gal synthesis in these truncation derivatives was monitored during sporulation in NSMP (**Figure 3A**, left), the P*_kinB_* (-55/+10)-*lacZ* derivative exhibited a higher level of β-Gal synthesis than the other truncation derivatives [P*_kinB_* (-95/+10), P*_kinB_* (-85/+10), P*_kinB_* (-75/+10), and P*_kinB_* (-65/+10)], which showed similar levels. When it was monitored upon decoyinine addition to the S6 cultures (**Figure 3A**, right), the basal level of β-Gal synthesis by the P*_kinB_* (-55/+10)-*lacZ* derivative before decoyinine addition was higher in comparison with those by the other derivatives. However, the positive stringent transcription control of P*_kinB_* was observed to be nearly the same level, approx.1.5-fold increase, for all the truncation derivatives, suggesting that the relief from *kinB* repression is not involved in this positive stringent transcription control. These overall results suggest that a binding site of a repressor or part of it is likely located in the P*_kinB_* region (nt -65/-55), which is responsible for *kinB* repression but not involved in positive stringent transcription control of P*_kinB_*.

Next, we introduced inner deletions [Δ10 (10-nt deletion, nt -64/-55) and Δ5 (5-nt deletion, nt -61/-57)] into the P*_kinB_* (-75/+10) region to confirm that the repressor-binding site is located between nt -65/-55. When β-Gal synthesis by the derivatives carrying each of the inner deletions [P*_kinB_* (-75/+10 Δ5) and P*_kinB_* (-75/+10 Δ10) was monitored together with that by strains carrying no inner deletion, P*_kinB_* (-75/+10) and P*_kinB_* (-55/+10) (**Figure 3B**), the inner deletion derivatives exhibited higher levels of β-Gal synthesis than that by the derivative without them [P*_kinB_* (-75/+10)] for sporulation in NSMP (**Figure 3B**, left) and in decoyinine-induced sporulation (**Figure 3B**, right). Moreover, the higher levels of β-Gal synthesis by these derivatives were in the order of P*_kinB_* (-75/+10 Δ5), P*_kinB_* (-75/+10 Δ10), P*_kinB_* (-55/+10), and P*_kinB_* (-75/+10). The differences could be attributed to newly created sequence variation of nt -65/-55 in these deletion derivatives, which might affect the binding of the assumed *kinB* repressor. However, nearly the same level of positive stringent transcription control was observed for these derivatives (**Figure 3B**, right). The overall deletion analysis (**Figure 3**) indicated that the inner deletion of Δ5 (AGGCG, nt -61/-57) disrupted a binding site or part of it for the assumed repressor for *kinB* transcription, which is not involved in positive stringent transcription control of P*_kinB_.*

### Identification of a Putative Binding Site of SinR for *kinB* Repression, and Involvement of SinR in Positive Stringent Transcription Control of P*_kinB_*

The *sinR* gene was isolated as a sporulation inhibition gene in multiple copies (Gaur et al., 1986). At first, we determined the sporulation percentages (%) during cultivation in NSMP medium and during cultivation in S6 after decoyinine addition. The sporulation percentages for strains 168 and FU1204 (*ΔsinR*) in NSMP were < 5 × 10^-5^% at T0, and 80 and 100% at T20, respectively. The sporulation percentages for strains 168 and FU1204 in decoyinine-induced sporulation were 0.4% and 1.5% at T0 (at decoyinine addition time) and 40% and 98% at T10. (The sporulation experiments were repeated at least three times. Representative values were presented. The standard deviations were less than 15% of the values shown.) Hence, the *ΔsinR* deletion tended to promote the sporulation, especially on cultivation in S6 medium with decoyinine.

**FIGURE 4:**
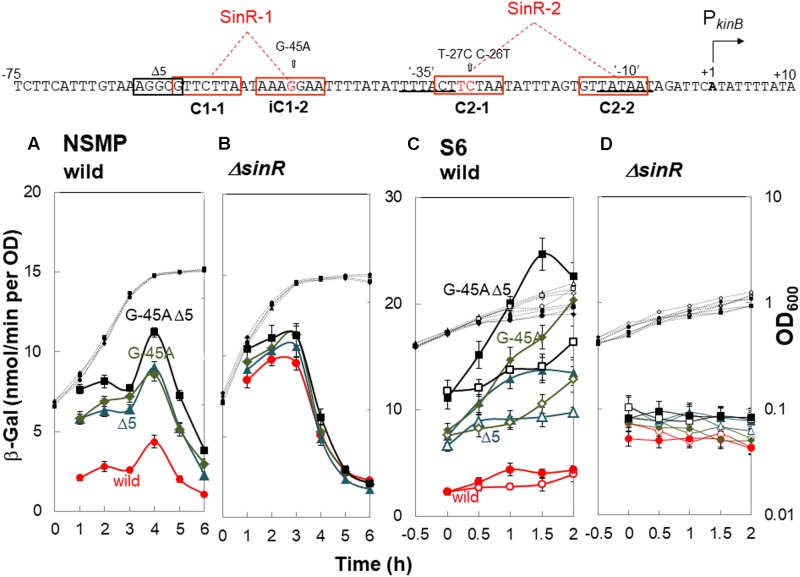
*In vivo* identification of SinR-1 for *kinB* repression by SinR. (Top) The nt sequence of the P*_kinB_* region (nt -75/+10) is shown, SinR-1 and SinR-2 being indicated. **(A,C)** β-Gal synthesis by strains FU1191 P*_kinB_* (-75/+10) (circles), FU1195 P*_kinB_* (-75/+10 Δ5) (triangles), FU1216 (P*_kinB_* (-75/+10 G-45A) (diamonds), and FU1217 P*_kinB_* (-75/+10 Δ5 G-45A) (squares) in the wild-type genetic background was monitored during sporulation in NSMP medium and after addition of decoyinine to S6 medium. **(B,D)** β-Gal synthesis by strains FU1210 [P*_kinB_* (-75/+10) *ΔsinR*] (circles), FU1224 [P*_kinB_* (–75/+10 Δ5) *ΔsinR*] (triangles), FU1218 [P*_kinB_* (-75/+10 G-45A) *ΔsinR*] (diamonds), and FU1219 [P*_kinB_* (-75/+10 Δ5 G-45A) *ΔsinR*] (squares) in the *ΔsinR* background was monitored during sporulation in NSMP medium and after addition of decoyinine to S6 medium.

A previous study involving a *lacZ*-fusion with the P*_kinB_* region (Dartois et al., 1996) suggested that *kinB* expression is repressed by SinR, and the substitution of guanine at nt -45 in the P*_kinB_* region with adenine resulted in relief from SinR repression. Thus, we constructed four strains each carrying P*_kinB_* (-75/+10), P*_kinB_* (-75/+10 Δ5), P*_kinB_* (-75/+10 G-45A), and P*_kinB_* (-75/+10 Δ5 G-45A), in the wild-type (*sinR*^+^) and *ΔsinR* genetic backgrounds. In the *sinR*^+^ strains cultivated in NSMP medium, the introduction of the inner deletion of Δ5 or the base substitution (G-45A) greatly and equally relieved the severe repression of *lacZ* expression observed in strain [P*_kinB_* (-75/+10)] without the deletion or substitution (**Figure 4A**). Moreover, the introduction of both Δ5 and G-45A gave further relief from the repression. In the *ΔsinR* strains cultivated in NSMP, the severe repression of the strain without Δ5 and G-45A as well as the residual repression observed in the Δ5 or G-45A strain were well relieved on the introduction of *ΔsinR* (**Figure 4B**).

For decoyinine-induced sporulation of the *sinR*^+^ background strains in S6 medium, Δ5 or G-45A equally well relieved the severe repression in strain P*_kinB_* (-75/+10) carrying no deletion or base substitution (**Figure 4C**). Also, it was completely relieved in strain P*_kinB_* (-75/+10) carrying both Δ5 and G-45A. In *ΔsinR* strains cultivated in S6 (**Figure 4D**), the levels of *lacZ* expression before decoyinine addition were nearly the same in strains P*_kinB_* (-75/+10) with and without Δ5 and (or) G-45A, indicating that the repression observed in strain P*_kinB_* (-75/+10) was well relieved on the introduction of *ΔsinR*. Surprisingly, positive stringent transcription control of P*_kinB_*, which is inducible through the addition of decoyinine, did not occur in any *ΔsinR* strain with and without Δ5 and (or) G-45A at all (**Figure 4D**). However, significant repression of *lacZ* expression was remained even in the genetic background of *ΔsinR*, as observed in **Figure 4D** as well as in **Figure 4B**. These results indicated that SinR is involved in positive stringent transcription control of P*_kinB_*.

A mild plateau of β-Gal synthesis around T0 and a clear decrease in it after T1 were observed in all *ΔsinR* strains for sporulation in NSMP (**Figure 4B**). We could not explain this sporulation phase-dependent variation of β-Gal synthesis without SinR regulation, because SinR repression of *kinB* transcription and positive stringent transcription control of P*_kinB_* did not occur in the *ΔsinR* strains during sporulation under the cultivation conditions (**Figures 4B,D**).

The consensus DNA binding sequence for SinR comprises a sequence (GTTCTYT) that can be found in inverted and tandem repeat arrangement/orientation and in a monomer state at SinR operator sites (Kearns et al., 2005; Chu et al., 2006; Colledge et al., 2011). Examination of the P*_kinB_* sequence around the Δ5 deletion and the G-45A substitution allowed us to identify a pair of SinR consensus sequences [C1-1 (nt -57/-51) and iC1-2 (-48/-42)] in an inverted repeat orientation (SinR-1 site), each consensus unit containing part of the Δ5 deletion or the G-45A substitution [**Figures 1, 4** (Top)]. Therefore, SinR-1 is most likely a SinR-binding site for *kinB* repression.

**FIGURE 5:**
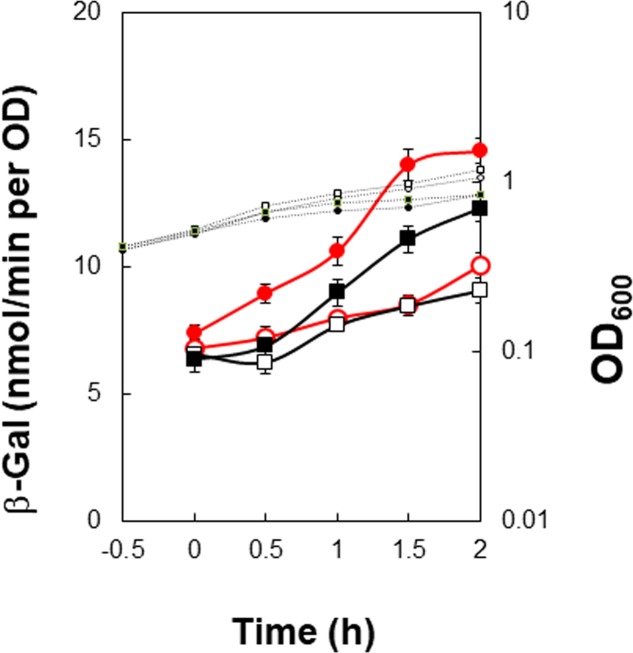
Partial decrease of positive stringent transcription control of P*_kinB_* induced by base substitutions (T-27C C-26T). β-Gal synthesis by strains FU1115 P*_kinB_* (–55/+10) (circles), and FU1249 P*_kinB_* (–55/+10 T-27C C-26T) (squares) was monitored after addition of decoyinine to S6 medium. The β-Gal monitoring was performed with quadruple replicates.

As described above, SinR is essential for positive stringent transcription control of P*_kinB_*_._ Examination of the sequences around the ‘-35’ and ‘-10’ regions revealed another pair of SinR consensus sequences [C2-1 (nt -29/-23) and C2-2 (-14/-8)] in a tandem repeat arrangement (SinR-2 site)[**Figures 1, 4** (Top)]. The C2-1 and C2-2 sequences partially overlap the ‘-10’ and ‘-35’ regions of P*_kinB_*, which might be possibly a SinR-binding site for positive stringent transcription control of P*_kinB_*. We attempted to isolate mutants of strain FU1115 [P*_kinB_* (-55/+10)-*lacZ*] that are defective in positive stringent transcription control of P*_kinB_*. We arbitrarily introduced nine base substitutions to the SinR-2 sequence (nt -29/-8) in the P*_kinB_* (-55/+10) region [A-17G, G-16A, T-15C, G-14A, (T-20C T-19C), (T-18C A-17G), (G-16A T-15C), (C-26T T-25C), and (C-26T T-27C)], and examined if the strength of each mutant P*_kinB_* is comparable to that of the wild-type, and if β–Gal synthesis under the control of the mutant P*_kinB_* is positively regulated after decoyinine addition. Thus, we found that only one mutant, FU1249 P*_kinB_* (-55/+10 C-26T T-27C) carrying the substitution in the C2-1 consensus sequence of SinR-2, synthesized β-Gal almost at the same level as wild-type strain FU1115, and exhibited partially impaired positive stringent transcription control in comparison with strain FU1115 (**Figure 5**). Although the other eight substitutions affected the P*_kinB_* strength, they did not affect positive stringent transcription control significantly (Supplementary Figure S1). The T-15C, G-14A, and (G-16A T-15C) mutations abolished the P*_kinB_* activity. The A-17G, (T-20C T-19), and (T-18C A-17G) mutations decreased it by several-fold, but did not affect positive stringent transcription control. The G-16A and (C-26T T-25C) mutations considerably enhanced the P*_kinB_* activity, but they did not affect positive stringent transcription control significantly. These results suggested that the C2-1 consensus sequence of SinR-2, where the C-26T T-27C substitution only affecting positive stringent transcription control of P*_kinB_* is located, is likely involved in its positive stringent transcription control.

### Examination of the Effects of *ΔsinR, ΔslrR*, and *ΔsinI* on *kinB* Repression and Positive Stringent Transcription Control of P*_kinB_*

**FIGURE 6:**
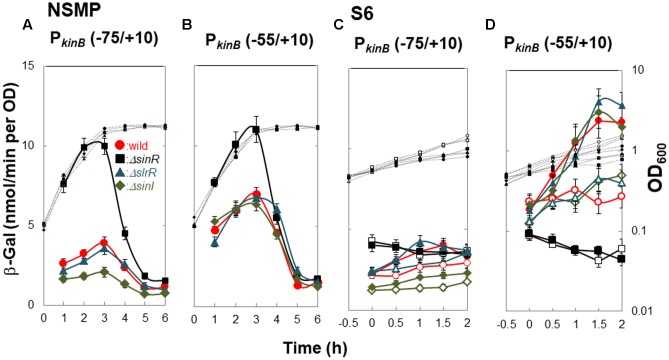
Effects of *ΔsinR*, *ΔslrR*, and *ΔsinI* on β-Gal synthesis under the control of P*_kinB_*. **(A,C)** β-Gal synthesis by strains FU1191 P*_kinB_* (–75/+10)(circles), FU1210 [P*_kinB_* (–75/+10) *ΔsinR*] (squares), FU1231 [P*_kinB_* (–75/+10) *ΔslrR*] (triangles), and FU1230 [P*_kinB_* (–75/+10) *ΔsinI*] (diamonds) was monitored during sporulation in NSMP medium and after the addition of decoyinine to S6 medium. **(B,D)** β-Gal synthesis by strains FU1115 P*_kinB_* (–55/+10)(circles), FU1206 [P*_kinB_* (–55/+10) *ΔsinR*] (squares), FU1238 [P*_kinB_* (–55/+10) *ΔslrR*] (triangles), and FU1237 [P*_kinB_* (–55/+10) *ΔsinI*] (diamonds) was monitored similarly.

*ΔsinR* relieved the repression of *kinB* transcription involving SinR-1, as described above (**Figure 4**). It also abolished positive stringent transcription control of P*_kinB_*. Thus, we examined the effects of SlrR (Kobayashi, 2008), a paralog of SinR, and SinI (Bai et al., 1993), an antagonist of SinR, on *kinB* repression and positive stringent transcription control of P*_kinB_*. We constructed *lacZ*-fusion strains P*_kinB_* (-75/+10) carrying *ΔslrR* or *ΔsinI*, and P*_kinB_* (-55/+10) carrying *ΔsinR*, *ΔslrR* or *ΔsinI*. As shown in **Figure 6A**, β-Gal synthesis by strain [P*_kinB_* (-75/+10) *ΔsinR*] was largely relieved from the repression in the wild-type strain P*_kinB_* (-75/+10) in sporulation in NSMP medium. *ΔslrR* did not relieve the repression, but *ΔsinI* further strengthened it. Thus, *kinB* repression by SinR seemed hard to be relieved in the absence of SinI. Moreover, *ΔsinR* also relieved mild repression by SinR which resulted from partial deletion of the C1-1 sequence in P*_kinB_* (-55/+10) (**Figure 6B**). Neither *ΔslrR* nor *ΔsinI* affected this mild repression.

Strains [P*_kinB_* (-75/+10) *ΔsinR*] and [P*_kinB_* (-55/+10) *ΔsinR*] exhibited neither the repression nor positive stringent transcription control of P*_kinB_* on decoyinine-induced sporulation in S6 (**Figures 6C,D**). Strain [P*_kinB_* (-75/+10) *ΔslrR*] exhibited no significant difference in either the repression or positive stringent transcription control in comparison to wild-type strain P*_kinB_* (-75/+10) (**Figure 6C**). *ΔsinI* did not affect positive stringent transcription control of P*_kinB_*_._ But, *lacZ* expression in strain [P*_kinB_* (-75/+10) *ΔsinI*] was most severely repressed, that is, this strain exhibited the lowest level of β-Gal synthesis before decoyinine addition (**Figure 6C**). On the other hand, the *lacZ* fusion strains [P*_kinB_* (-55/+10) *ΔsinI*] and [P*_kinB_* (-55/+10) *ΔslrR*] exhibited almost the same level of the repression and positive stringent transcription control of P*_kinB_* as the wild-type strain P*_kinB_* (-55/+10) (∼1.5-fold increase) (**Figure 6D**). The overall results indicated that SinI deficiency causes stronger SinR-dependent repression and reduces derepression, but SlrR is not involved in the repression, and also indicated that SinR is involved in positive stringent transcription control of P*_kinB_*, but SinI and SlrR are not.

### EMSA Analysis of SinR Binding with Probes Carrying Deletion and Base-Substitution That Affect *kinB* Regulation *in Vivo*

**FIGURE 7:**
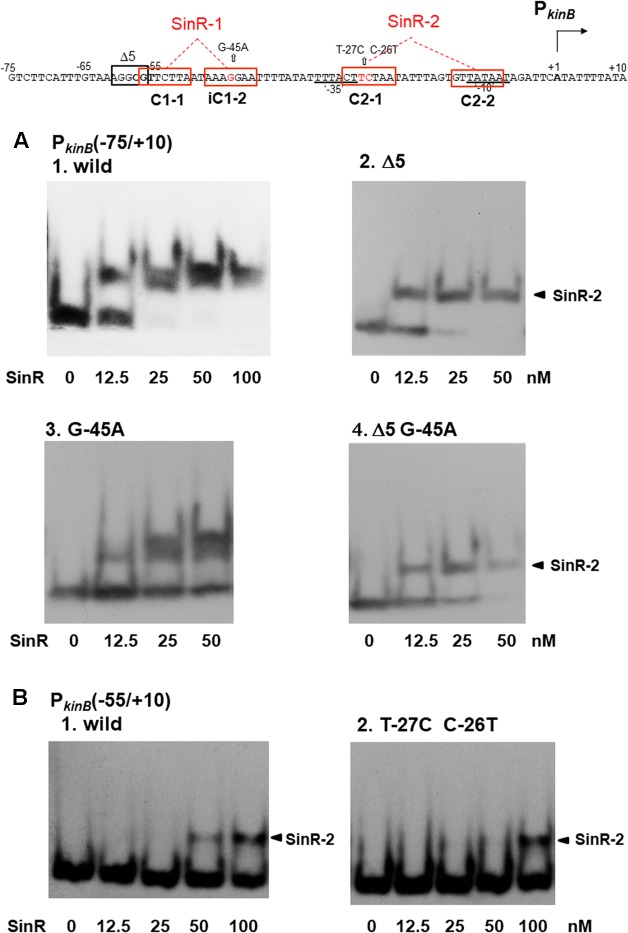
Electrophoretic mobility shift assay (EMSA) analysis of SinR binding to the P*_kinB_* region carrying deletion or base substitutions that affect β-Gal synthesis on *lacZ*-fusion analysis. (Top) The nt sequence of the P*_kinB_* region (nt –75/+10) is shown, SinR-1 and SinR-2 being indicated. **(A) [1]** EMSA results for SinR-binding to the P*_kinB_* (–75/+10) probe in a 5% non-denaturing polyacrylamide gel. Increasing amounts of SinR (0, 12.5, 25, 50 nM) were used. (nM was calculated as the SinR monomer.) Upper and lower bands resulting from SinR-binding to the probe appeared. **[2,3,4]** EMSA results using the P*_kinB_* (–75/+10) probe carrying the Δ5 deletion, the G-45A base substitution, and Δ5 and G-45A, respectively. Hence, these mutant probes carry only an intact SinR-2. The arrowheads **[2,4]** indicate the position of the shifted band resulting from SinR binding to SinR-2. **(B)** EMSA results with the P*_kinB_* (-55/+10) probe carrying only SinR-2 consisting of C2-1 and C2-2. **[1]** EMSA results using the P*_kinB_* (–55/+10) probe (wild-type). **[2]** EMSA results using the P*_kinB_* (–55/+10) probe carrying the T-27C C-26T substitution in the C2-1 consensus sequence. DNA probes were prepared by use of primer pair and template DNA listed in Supplementary Tables S1, S2-2.

On *lacZ* fusion analysis using Δ*sinR* as well as Δ5, G-45A, and C-26T T-27C, SinR was found to be responsible not only for *kinB* repression involving SinR-1 consisting of C1-1 and iC1-2 (**Figure 4**), but also for positive stringent transcription control of P*_kinB_* probably involving SinR-2 consisting of C2-1 and C2-2 (**Figures 4, 5**). On EMSA analysis using the probes carrying Δ5 and G-45A, and C-26T T-27C, we found that these mutations actually affected *in vitro* SinR binding to SinR-1 and to SinR-2, respectively, as follows (**Figure 7**).

As shown in **Figure 7A-1**, the wild-type P*_kinB_* (-75/+10) probe gave the two closely located bands on EMSA, which likely resulted from SinR binding to SinR-1 and SinR-2. The upper band is invisible at 12.5 nM SinR, and visible at 25 nM with the P*_kinB_* (-75/+10) probe carrying the G-45A substitution, likely resulting from approximately 2-fold less binding affinity to SinR-1 (**Figure 7A-3**). This band disappeared with the probe carrying the Δ5 deletion or Δ5 and G-45A (**Figures 7A-2,-4**), suggesting that SinR cannot bind to SinR-1 if part of the C1-1 sequence is deleted by Δ5.

As described above, **Figure 4C** shows that in the wild-type cells with P*_kinB_* (-75/+10) during cultivation in S6 medium, as well as in those with P*_kinB_* (-75/+10) possessing Δ5 (and G-45A), approximately 1.5-fold positive stringent transcription control of P*_kinB_* steadily occurred, regardless of the level of *kinB* repression before decoyinine addition. The same level of positive stringent transcription control was also observed in the cells of a series of truncation and deletion derivatives of the P*_kinB_* region (nt -95/+10 to -55/+10) that exhibited different levels of *kinB* repression (**Figure 3**). These results suggested that SinR simultaneously binds to both SinR-1 and SinR-2 to form a larger complex than that on SinR binding to SinR-1 or SinR-2. Nevertheless, a more slowly migrating band other than the two closely located bands did not exist (**Figure 7A**). Thus, the closely located upper and lower bands were considered to probably result from simultaneous SinR binding to SinR-1 and -2, and from SinR binding to SinR-1 or SinR-2, respectively.

The C-26T T-27C substitution located in C2-1 partially affected the positive stringent transcription control (**Figure 5**). EMSA with the probe of P*_kinB_* (-55/+10) deleting part of C1-1 of SinR-1 gave only a shifted band most likely resulting from SinR binding to SinR-2 (**Figure 7B**). SinR binding affinity to SinR-2 with the probe carrying the C-26T T-27C substitution (**Figure 7B-2**) was significantly less than that of the wild-type P*_kinB_* (-55/+10) (**Figure 7B-1**).

### EMSA Analysis for SinR Binding to SinR-1 and SinR-2 Using Deleted and Mutated Probes

**FIGURE 8:**
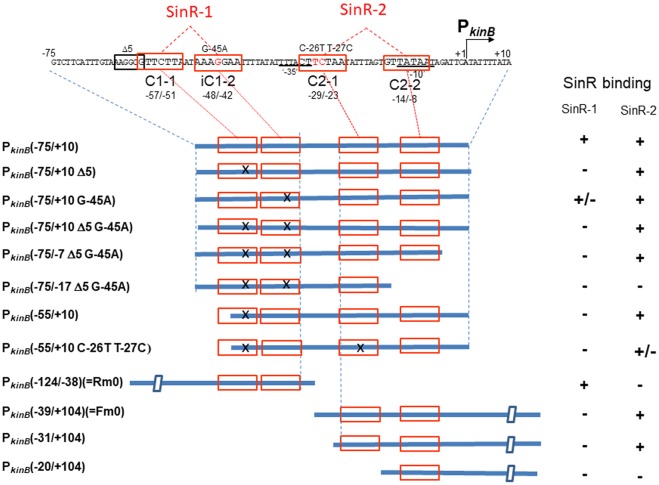
Deletion analysis to localize SinR-binding sites (SinR-1 and SinR-2) by EMSA. (Top) The nt sequence of the P*_kinB_* region (nt –75/+10), where SinR-1 (C1-1 and iC1-2) and SinR-2 (C2-1 and C2-2) are localized, is shown. The EMSA probes cover various P*_kinB_* regions indicated by thick blue bars where SinR consensus sequences (red boxes) are shown; X means a defective consensus sequence.

**Figure 8** shows the arrangement of SinR-binding sites (SinR-1 and SinR-2), and illustrates the covering of the P*_kinB_* region by various probes for EMSA. The SinR binding ability to SinR-1 and (or) SinR-2 of the probes (+, +/- or -) is given in the right columns. SinR bound to SinR-1 and SinR-2 of the P*_kinB_* (-75/+10) probe, but it did not bind to SinR-1 of its mutant derivatives (**Figure 7A**). SinR bound to SinR-2 of the P*_kinB_* (-55/+10) probe, but it only partially bound to its mutant derivative (**Figure 7B**). SinR bound to SinR-1 of P*_kinB_* (-124/–38) (Rm0) and SinR-2 of P*_kinB_*-39/+104) (Fm0) (Supplementary Figure S3). It is particularly notable that the EMSA results that SinR bound to the P*_kinB_* (-75/-7 Δ5 G-45A) probe but not to the P*_kinB_* (-75/-17 Δ5 G-45A) probe (Supplementary Figures S2-1,-2) indicated that C2-2 is essential for SinR binding to SinR-2. (The migration rate of the very faint band observed in Supplementary Figure S2-2 was nearly half of that observed in Supplementary Figure S2-1, which might have resulted from binding of the SinR monomer to C1-1.) Moreover, the EMSA results that SinR bound to the P*_kinB_* (-31/+104) probe but not to the P*_kinB_* (-20/+104) probe (Supplementary Figures S2-3,-4) indicated that C2-1 is indispensable for SinR binding to SinR-2. (A very faint band observed in Supplementary Figure S2-4 is unknown.) These EMSA results involving deleted and base-substituted probes (**Figure 8**) allowed us to conclude that both C1-1 and iC1-2 of SinR-1 are necessary for SinR binding to SinR-1, whereas both C2-1 and C2-2 are necessary for SinR binding to SinR-2.

It should be noted that EMSA analyses involving the probes of P*_kinB_* (-75/+10) and P*_kinB_* (-75/-7) carrying Δ5 G-45A gave an apparent equilibrium dissociation constant (*K*_d_) of approximately 10 nM for SinR binding to SinR-2 (**Figure 7A** and Supplementary Figure S2-1), but EMSA involving P*_kinB_* (-55/+10), P*_kinB_* (-31/+104), and P*_kinB_* (-39/+104) (Fm0) probes gave *K*_d_ of more than 100 nM for SinR binding to SinR-2 (**Figure 7B-1** and Supplementary Figure S3-2). This finding implies that an unidentified sequence upstream of nt -55 might function to enhance SinR binding to SinR-2 without its binding to SinR-1. This unknown enhancement of SinR binding to SinR-2 remains to be studied.

**FIGURE 9:**
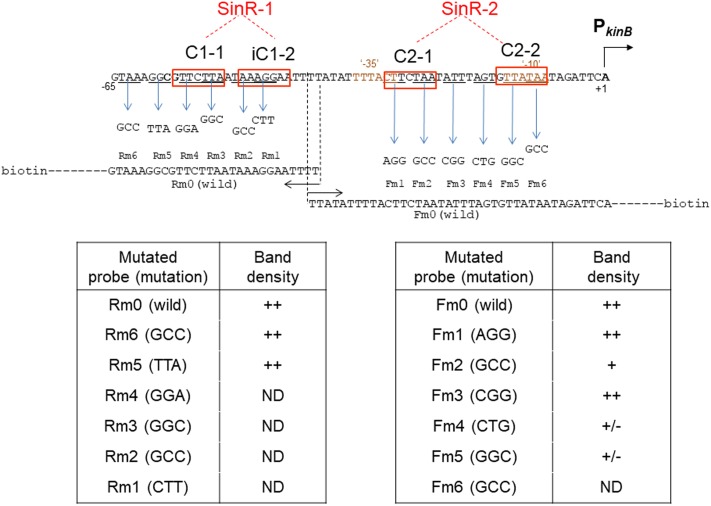
Electrophoretic mobility shift assay analysis of SinR binding to the mutant P*_kinB_* probes. The wild-type probes [P*_kinB_* (–124/–38) ( = Rm0) and P_kinB_ (–39/+104) ( = Fm0)], as well as the mutant ones carrying the respective three-base substitutions in the SinR-1 site (Rm1, Rm2, Rm3, Rm4, Rm5, and Rm6) and in the SinR-2 site (Fm1, Fm2, Fm3, Fm4, Fm5, and Fm6) were prepared using the wild-type and mutant primer pairs and a DNA template (Supplementary Tables S1, S2-2).

**Figure 9** summarizes the results of EMSA analyses involving a series of three-base substituted PCR probes to determine which parts of SinR-1 and SinR-2 sequences are necessary for SinR binding. For EMSA to determine which part of the SinR-1 is necessary for SinR binding, the mutant probes (Rm6, Rm5, Rm4, Rm3, Rm2, and Rm1) and the wild-type one (Rm0) were used, as illustrated on the left side of **Figure 9**. The EMSA results as to Rm0 and Rm1 to Rm6 are shown in the upper panel of Supplementary Figure S3-1. The relative densities of the shifted bands of the mutant probes [++, +, +/-, and ND (not detected)] to that of the wild-type (++) as to their binding to SinR-1 are arbitrary given in the lower left column from their vision (Supplementary Figure S3-1). The base-substitutions within C1-1 and iC1-2 of SinR-1 (Rm4, Rm3, Rm2, and Rm1) almost completely abolished the shifted band, whereas those upstream of C1-1 (Rm6 and Rm5) did not diminish the band density. The results indicated that both C1-1 and iC1-2 are essential for SinR-binding to SinR-1.

For EMSA of SinR binding to SinR-2, the mutant probes (Fm1, Fm2, Fm3, Fm4, Fm5, and Fm6) and the wild-type one (Fm0) were used, as illustrated on the right side of **Figure 9**. The EMSA data as to Fm0 and Fm1 to Fm6 are shown in the upper panel of Supplementary Figure S3-2. The relative band densities of the shifted bands of the mutant probes to that of the wild-type as to their binding to SinR-1 [++, +, +/-, and ND] are given in the lower right column. The base-substitutions in C2-1 (Fm2) only partially affected SinR binding to SinR-2, but those in C2-2 (Fm5) considerably affected it. Besides, the substitution (Fm4) immediately upstream of C2-2 partially affected it.

EMSA analysis with the mutant probes (**Figure 9**) indicated that SinR-binding to SinR-2 only partially requires C2-1 but it well requires C2-2. The EMSA results with deleted probes as described above (**Figure 8** and Supplementary Figure S2) suggested that both C2-1and C2-2 are likely essential for SinR binding to SinR-2. This inconsistency might reflect the difference between the three-base substitution in C-2-1 (**Figure 9**) and its complete elimination (**Figure 8**). However, the role of AGT just upstream of C2-2 in SinR binding to SinR-2 is unknown. These EMSA results suggested that both C2-1 and C2-2 are likely necessary for SinR binding to SinR-2, although C2-1 might not be so strictly required in comparison with C2-2. Thus, the SinR binding site (SinR-2) likely comprises the two SinR consensus of C2-1 and C2-2 sequences in a tandem arrangement, which partially overlap the ‘-35’ and ‘-10’ regions of P*_kinB_*, respectively.

Lastly, the EMSA data (Supplementary Figures S3-1,-2, upper panels) as to the wild-type and mutant probes (Rm0, Rm2, Rm4, Rm5, Fm0, and Fm5) were confirmed by EMSA with the gradient of the SinR concentration (Supplementary Figures S3-1,-2, lower panels); the probes (Rm4, Rm2, and Fm5) whose three-base substitutions (GGA, GGC, and GGC) are located within C1-1, iC1-2 and C2-2, respectively. *K*_d_ for SinR binding to Rm0 was approximately 50 nM, whereas *K*_d_ for SinR binding to Fm0 was more than 400 nM (Supplementary Figures S3-1,-2). SinR did not bind to SinR-1 of Rm2 and Rm4 at 200 nM of the SinR concentration and it did not bind to SinR-2 of Fm5 at 800 nM, clearly confirming that C1-1 and iC1-2, and C2-2 are necessary for SinR binding to SinR-1 and SinR-2, respectively.

The overall EMSA analyses clearly indicated that SinR binds to SinR-1 consisting of C1-1 and iC1-2 for transcription repression of P*_kinB_* and it binds to SinR-2 consisting of C2-1 and C2-2 for its positive stringent transcription control.

### EMSA Analysis of the Binding of SinR and RNA Polymerase (RNAP) to the P*_kinB_* Region

**FIGURE 10:**
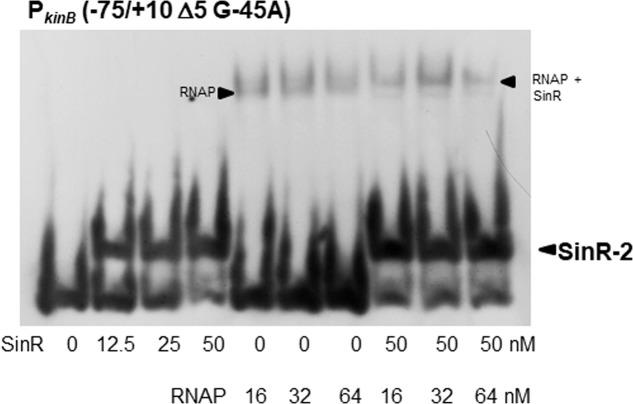
EMSA analysis of the binding of SinR and RNA polymerase (RNAP) to the P*_kinB_* region. The P*_kinB_* (–75/+10 Δ5 and G-45A) probe was used for EMSA. The slightly lower and upper arrowheads denote the positions of the complexes of SinR and the probe, and of RNAP, SinR and the probe, respectively.

We found that SinR-2 consists of C2-1 and C2-2 in tandem arrangement, which is likely involved in positive stringent transcription control of P*_kinB_*. C2-1 and C2-2 partially overlap the ‘-35’ and ‘-10’ regions of P*_kinB_* (**Figure 1**), so it was expected that SinR might bind to SinR-2 to form a transcription initiation complex of SinR, RNAP, and P*_kinB_* to exert its positive stringent transcription control. As shown in **Figure 10**, the electrophoretic band of a complex of the P*_kinB_* probe and RNAP appeared to shift to a slightly slower position when SinR was further added. This implies that a positively regulated stringent promoter such as P*_kinB_* might form a transcription initiation complex with SinR and RNAP for its positive stringent transcription control.

## Discussion

ppGpp is synthesized by the RelA protein associated with ribosomes upon amino acid starvation (Fujita et al., 2012). In the case of *E. coli*, the target of ppGpp is RNAP, stringent genes being regulated positively and negatively, depending on their specific promoter sequences. In contrast, the ppGpp target is GMP kinase in *B. subtilis*, the *in vivo* GTP concentration being reduced (Kriel et al., 2012). The GTP concentration also decreased upon addition of decoyinine, an inhibitor of GMP synthase (Tojo et al., 2008). The GTP decrease reciprocally results in an ATP increase via feedback regulation (Tojo et al., 2010, 2013). The transcription initiation of negative stringent control genes such as *rrn*, *ptsG*, and *pdhA*, whose transcription initiation base is guanine, is reduced upon a GTP decrease, whereas that of positive stringent control genes such as *ilvB*, *pycA*, *kinB*, and *kinA*, whose transcription initiation base is adenine, is enhanced upon an ATP increase (Krásný and Gourse, 2004; Krásný et al., 2008; Tojo et al., 2008, 2010, 2013).

Decoyinine induces sporulation of *B. subtilis* cells exponentially growing in the presence of rapidly metabolizable carbon, nitrogen, and phosphate sources (Mitani et al., 1977). It is known that the stringent response also induces sporulation (Ochi et al., 1981, 1982). Recently, decoyinine was found to induce positive stringent transcription control of the *kinB* gene encoding a trigger of sporulation (Tojo et al., 2013), which might be the reason why decoyinine induces sporulation. The *lacZ*-fusion analysis using the mutant cells carrying the A+1G substitution (**Figure 2**) disclosed that positive stringent transcription control has a larger contribution to *kinB* expression in decoyinine-induced sporulation in minimal S6 medium than in sporulation in nutrient NSMP medium. Both *kinB* repression by SinR and SinR-dependent positive stringent transcription control of P*_kinB_* simultaneously occur, as inferred from that approx. 1.5-fold positive stringent transcription control of P*_kinB_* was constantly and steadily observed after decoyinine addition to the S6 culture, regardless of the level of *kinB* repression before decoynine addition (**Figures 3, 4**).

The *sinR* strain exhibits the sporulation-deficient phenotype when present in multiple copies (Gaur et al., 1986). The *ΔsinR* strain sporulated a little bit better than the wild-type strain. The *kinB* gene was repressed by SinR (Dartois et al., 1996) (**Figure 4**). SinR was also involved in positive stringent transcription control of P*_kinB_* (**Figures 4, 5**). SinI is an antagonist of SinR (Bai et al., 1993; Chai et al., 2008; Chu et al., 2008), which is induced by Spo0A∼P (Shafikhani et al., 2002; Lopez et al., 2009). SinI induced during sporulation initiation eventually inhibits SinR, leading to relief of *kinB* repression through SinR detachment from its binding site. Thus, SinI deficiency resulted in stronger SinR-dependent repression and reduced derepression (**Figure 6**). SrlR, a protein homologous to SinR (Kobayashi, 2008), was unlikely involved in the relief from this SinR repression. Furthermore, neither SinI nor SrlR was involved in its positive stringent transcription control (**Figure 6**).

It should be noted that the relief from *kinB* repression caused by SinR, presumably mediated by SinI, is supposed to be quite insufficient for sporulation to proceed, as observed for sporulation in NSMP and for decoyinine-induced sporulation in the wild-type *sinR*^+^ genetic background (**Figures 3, 4**). Thus, in the wild-type SinR^+^ cells, the limited level of derepression of *kinB* from SinR repression by SinI induced by Spo0A∼P and significant induction of SinR-dependent positive stringent transcription control of P*_kinB_* upon stringent response cooperatively induce effective sporulation. It is inferred from the results (**Figures 4, 6**) that the level of *kinB* expression on sporulation of the wild-type strain is likely lower than that on sporulation of the Δ*sinR* strain even if positive stringent transcription control is blocked by Δ*sinR*. This might be the reason why the Δ*sinR* strain sporulated a little bit better than the wild-type strain.

Examination of the sequence of the P*_kinB_* region revealed two SinR binding sites (SinR-1 and SinR-2), i. e. a pair of SinR consensus sequences (C1-1 and iC1-2) in an inverted orientation, and another pair of SinR ones (C2-1 and C2-2) in a tandem arrangement, respectively (**Figure 1**). Such SinR-binding motifs consisting of a pair of SinR consensus sequences in an inverted orientation and a tandem arrangement are often observed in the promoter regions of the operons involved in biofilm formation such as *espA-O* (Kearns et al., 2005) and *tapA-sipW-tasA* (Chu et al., 2006). *In vivo* deletion and base substitution analyses of SinR-1 for *kinB* repression (**Figures 3, 4**) and EMSA using various deleted and mutated probes (**Figures 7–9**) revealed that both C1-1 and iC1-2 are necessary for *kinB* repression and SinR binding to SinR-1. Moreover, the base substitution in C2-1, which was involved in positive stringent transcription control of P*_kinB_* (**Figure 5**), also affected SinR binding to SinR-2 (**Figure 7**). EMSA using various deleted and mutated probes (**Figures 8, 9**) suggested that both C2-1and C2-2 are necessary for *SinR* binding to SinR-2.

The *sinR* deletion (*ΔsinR*) abolished positive stringent transcription control of P*_kinB_* (**Figure 4**). *lacZ*-fusion analysis of the other stringently-controlled promoters (unpublished observation by S. Nii and Y. Fujita) indicated that *ΔsinR* also abolished the positive stringent transcription control of P*_ilvB_*, P*_pycA_*_,_ and P*_kinA._* Interestingly, *ΔsinR* did not affect the negative stringent transcription control of P*_ptsG_* and P*_pdhA_*. This observation suggested that positive stringent transcription control involves SinR, but negative stringent transcription control does not involve it. EMSA analyses (**Figures 7–9**) showed that the P*_kinB_* region actually possesses an SinR-binding site (SinR-2), i. e. a pair of C2-1 and C2-2 sequences partially overlapping the ‘-35’ and ‘-10’ regions, respectively, which is likely involved in positive stringent transcription control of P*_kinB_* (**Figure 5**). Furthermore, EMSA indicated that a complex of RNAP, SinR, and P*_kinB_* for transcription initiation is likely formed, implying that SinR might be involved in transcription initiation of positively controlled stringent genes (**Figure 10**). Detailed investigation of the molecular mechanism involving SinR underlying positive stringent transcription control is in progress.

## Author Contributions

YF, SN, and KH performed *in vivo* study of *kinB* regulation by SinR in *B. subtilis* from April 2015 to March 2017 in Fukuyama University. YF moved from Fukuyama University to Tokai University April 2017. YF and MO (Tokai University) performed *in vitro* study of this work from April 2016 to July 2017.

## Conflict of Interest Statement

The authors declare that the research was conducted in the absence of any commercial or financial relationships that could be construed as a potential conflict of interest.

## Footnotes

1 http://subtiwiki.uni-goettingen.de/
