# Supplementary material for: Dual Regulation of Bacillus subtilis kinB Gene Encoding a Sporulation Trigger by SinR through Transcription Repression and Positive Stringent Transcription Control
Source: Front Microbiol. 2017 Dec 13;8:2502. doi: 10.3389/fmicb.2017.02502 (PMC5733473; doi:10.3389/fmicb.2017.02502)
Supplement: Supplementary file 1 [file Presentation_1.PDF]

Dual regulation of *Bacillus subtilis kinB* gene encoding a sporulation trigger by SinR through transcription repression and positive stringent transcription control

Supplemental material

Fig. S1 (Monitoring of β-Gal synthesis in strains carrying the base substitutions in the SinR-2 region), Fig. S2 (EMSA results with the gradient of the SinR concentration showing SinR-binding ability to truncated P_{kinB} probes), Fig. S3 (EMSA results using the mutant probes), Table S1 (Primer pair and template DNA for preparation of EMSA probes), and Table S2 (Sequence of primers for PCR).

Figures (supplementary)

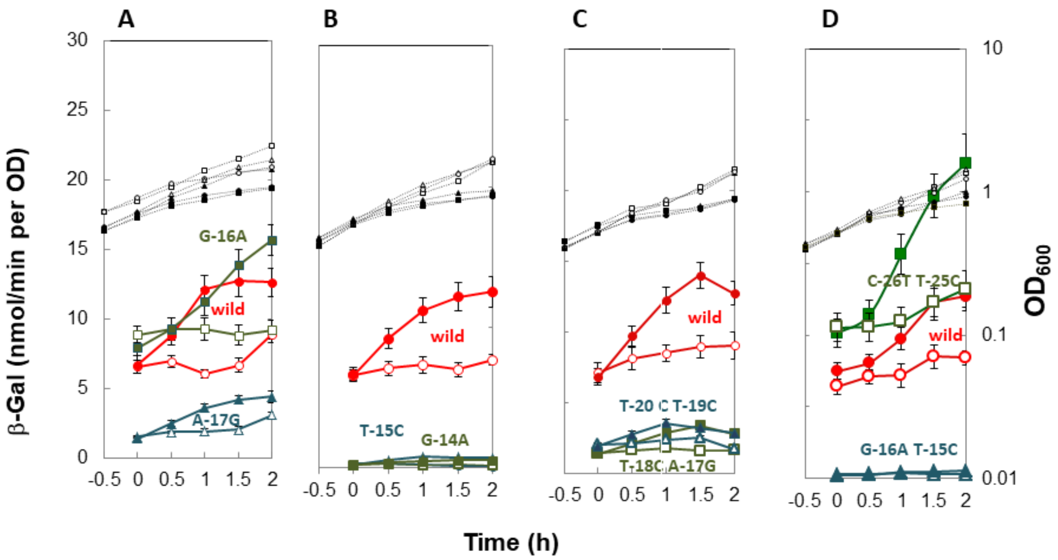

Fig. S1 **Monitoring of β–Gal synthesis in strains carrying the base substitutions in the SinR-2 region**. β-Gal synthesis by strains FU1241 P_{kinB} (-55/+10 A-17G) (triangles) and FU1242 P_{kinB} (-55/+10 G-16A) (squares) (**A**), by strains FU1243 P_{kinB} (-55/+10 T-15C) (triangles) and FU1244 P_{kinB} (-55/+10 G-14A) (squares) (**B**), by strains FU1245 P_{kinB} (-55/+10 T-20C T-19C) (triangles) and FU1246 P_{kinB} (-55/+10 T-18C A-17G) (squares) (**C**), and by stains FU1247 P_{kinB} (-55/+10 G-16A T-15C) (triangles) and FU1248 P_{kinB} (-55/+10 C-26T T-25C)(squares) (**D**) was monitored after addition of decoyinine to S6 medium. β-Gal synthesis by wild-type strain FU1115 P_{kinB} (-55/+10) (circles) was monitored together with each set of the mutants.

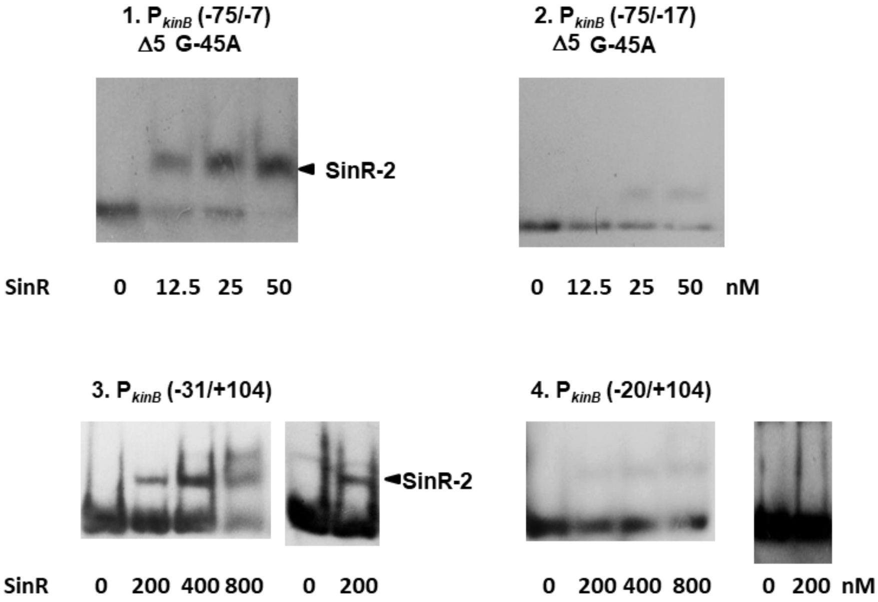

Fig. S2. **EMSA results with the gradient of the SinR concentration to examine SinR-binding ability to truncated P_{kinB} probes.** Refer to Fig. 8 as to the P_{kinB} regions covered by various P_{kinB} probes. EMSA results of the SinR binding to SinR-2 of the P_{kinB} (-75/-7) probe carrying Δ5 and G-45A **(1)**, to the P_{kinB} (-75/-17) probe carrying Δ5 and G-45A **(2),** to SinR-2 of P_{kinB}(-31/+104) **(3)**, and to P_{kinB}(-20/+104) **(4)** are shown.

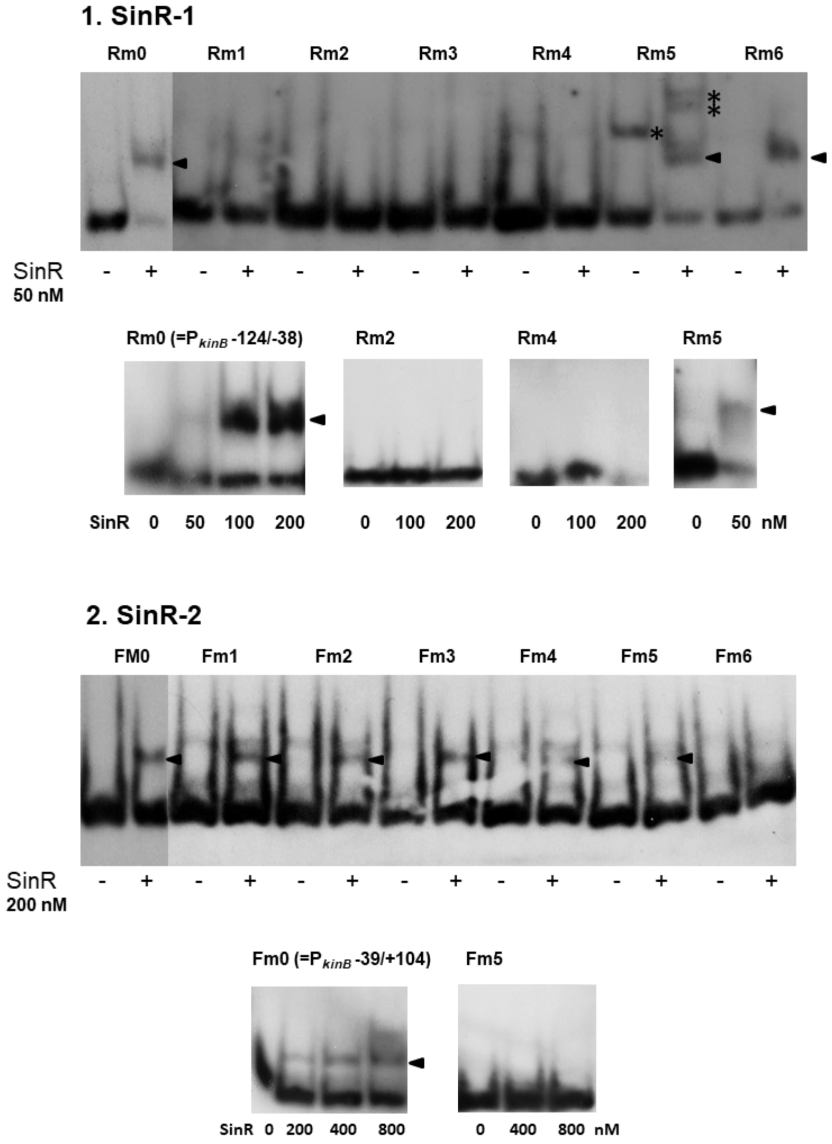

Fig. S3. **EMSA results using the mutant probes. (1)** The upper panel shows the EMSA results using the wild-type and mutant P_{kinB}(-124/-38) (Rm0 and Rm1-6) probes as to SinR binding to SinR-1. The lower panels show the EMSA results of Rm0, Rm2, and Rm4 with the gradient of the SinR concentration. The Rm5 lanes of the lower panel (SinR, 0 and 50 nM) do not contain unknown extra-bands, which are indicated with asterisks in the Rm5 lanes of the upper panel. Arrowheads indicate the shifted bands. **(2)** The upper panel shows the EMSA results using the wild-type and mutant P_{kinB}(-39/+104) probes (Fm0 and Fm1-6) as to SinR binding to SinR-2. The lower panels show the EMSA results of Fm0 and Fm5 with the gradient of the SinR concentration.

Table S1. Primer pair and template DNA for preparation of EMSA probes

DNA probe	Primer pair	Template DNA strain
P_{kinB}(-75/+10)	Fb0/R0	FU1191
P_{kinB}(-75/+10 Δ5)	Fb0/R0	FU1195
P_{kinB}(-75/+10 G-45A)	Fb0/R0	FU1216
P_{kinB}(-75/+10 Δ5 G-45A)	Fb0/R0	FU1217
P_{kinB}(-55/+10)	Fb0/R0	FU1115
P_{kinB}(-55/+10 T-27C C-26T)	Fb0/R0	FU1249
P_{kinB}(-75/-7 Δ5 G-45A)	Fb0/R1	FU1217
P_{kinB} (-75/-17 Δ5 G-45A)	Fb0/R2	FU1217
P_{kinB}(-124/-38) (=Rm0)	Fb1/Rm0	168
P_{kinB}(-39/+104) (=Fm0)	Fm0/Rb1	168
P_{kinB}(-31/+104)	F1/Rb1	168
P_{kinB}(-20/+104)	F2/Rb1	168
Rm1	Fb1/Rm1	168
Rm2	Fb1/Rm2	168
Rm3	Fb1/Rm3	168
Rm4	Fb1/Rm4	168
Rm5	Fb1/Rm5	168
Rm6	Fb1/Rm6	168
Fm1	Fm1/Rb1	168
Fm2	Fm2/Rb1	168
Fm3	Fm3/Rb1	168
Fm4	Fm4/Rb1	168
Fm5	Fm5/Rb1	168
Fm6	Fm6/Rb1	168

Table S2. Sequence of primers for PCR

1. Primers for strain construction

Primer	Sequence
F75c	gcgcgcgctctagatcttcatttgtaaaggcgt
F55c	aagctgtcaaacatgagaattct
R10c1	gtgggatcctataaaatatgaatctattataa
R10c2	gtgggatcctataaaatatgaatctattataacac
R10c3	gcattagtgtatcaacaagctgg
F04a	taccagtccgacatgaaaaaggat
R04b	gcactatcaacacactcttaagtgtcatcaccttccttgtgatat
F04c	cttaagagtgtgttgatagtgc
R04d	ctagggacctctttagctcc
F04e	ggagctaaagaggtccctagtgcctgagcagaggcactaa
R04f	gcagcatttaacgacattaaatcaaa
F16a	gcgcgcgctctagatcttcatttgtaaaggcgttcttaata
R16b	aatataaaattcttttattaagaacgcct
F16c	ttcttaataaaAgaattttatatt
F17	gcgcgcgctctagatcttcatttgtaattcttaataaaAgaattttatattttacttcta
R17	gtgggatcctataaaatatgaatctattataacactaaat
F82	gcgcgcgctctagaatagctgtaaacgccttta
F90	gcgcgcgctctagataaaggcgttcttaataaag
F92	gcgcgcgctctagacgcctttacgtcttcatt
R93	gtgggatcctataaaatacGaatctattataa
F95	gcgcgcgctctagatcttcatttgtaattcttaataaaggaattttatatttta
F96	gcgcgcgctctagatcttcatttgcttaataaaggaattttatattttac
R41b	tgaatctattataacacCaaatattaaag
F41c	cttctaatatttGgtgttataatagattca
R42b	tgaatctattataacaTtaaatattagaag
F42c	cttctaatatttaAtgttataatagattca
R43b	tgaatctattataacGctaaatattagaag
F43c	cttctaatatttagCgttataatagattca
R44b	tgaatctattataaTactaaatattagaag
F44c	cttctaatatttagtAttataatagattca
R45b	tgaatctattataacactaGGtattagaag
F45c	cttctaataCCtagtgttataatagattca
R46b	tgaatctattataacacCGaatattagaag
F46c	cttctaatattCGgtgttataatagattca

R47b	tgaatctattataacGTtaaatattagaag
F47c	cttctaatatttaACgttataatagattca
R48b	taacactaaatattGAaagtaaaatataaa
F48c	tttatattttacttTCaatatttagtgtta
R49b	taacactaaatattaAGagtaaaatataaa
F49c	tttatattttactCTtaatatttagtgtta

The upper case bases are the introduced ones to construct the mutants.

2. Primers for preparation of EMSA probes

Primer	Sequence
Fb0	biotin-aagctgtcaaacatgagaattct
Fb1	biotin-gccgcatcaaagccgcattatcgt/
F1	tacttctaatatttagtgttataatagat
F2	tttagtgttataatagattcatatttt
R0	gcattagtgtatcaacaagctgg
R1	attataacactaaatattagaa
R2	taaatattagaagtaaaatataaaa
Rb1	biotin-gccaaaacacttggtaaagaagaataggaaac
Rm0	aaaattcctttattaagaacgcctttac
Rm1	aaaattAAGttattaagaacgcctttac
Rm2	aaaattcctGGCttaagaacgcctttac
Rm3	aaaattcctttatGCCgaacgcctttac
Rm4	aaaattcctttattaaTCCcgcctttac
Rm5	aaaattcctttattaagaaTAAtttac
Rm6	aaaattcctttattaagaacgcctGGCcaaat
Fm0	ttatattttacttctaatatttagtgtta
Fm1	ttatattttaAGGctaatatttagtgtta
Fm2	ttatattttacttcGCCtatttagtgtta
Fm3	ttatattttacttctaatCGGtagtgtta
Fm4	ttatattttacttctaatatttCTGgttataa
Fm5	ttatattttacttctaatatttagtgGGCtaatag
Fm6	ttatattttacttctaatatttagtgttaGCCtagattc

The upper case bases are the introduced ones to prepare the mutant probes.
